# Association of deep vein thrombosis with poststroke epilepsy after acute ischemic stroke: a multicenter retrospective cohort study

**DOI:** 10.3389/fneur.2026.1920544

**Published:** 2026-09-25

**Authors:** Erxinxian Bei, Runzhui Lin, Le Zhao, Yong He, Tieshi Zhu

**Affiliations:** 1 Department of Neurology, Haikou Affiliated Hospital of Central South University Xiangya School of Medicine, Haikou, China; 2 Department of Hepatobiliary, Pancreatic and Splenic Surgery, Second Affiliated Hospital of Shantou University Medical College, Shantou, China; 3 Department of Medical Affairs, Central Hospital of Guangdong Provincial Nongken, Zhanjiang, Guangdong, China; 4 Department of Neurology, Liuyang Jili Hospital, Changsha, Hunan, China; 5 Department of Neurology, Zhanjiang Central Hospital, Guangdong Medical University, Zhanjiang, Guangdong, China

**Keywords:** acute ischemic stroke, deep vein thrombosis, poststroke epilepsy, risk stratification, stroke severity

## Abstract

**Background:**

Deep vein thrombosis (DVT) and poststroke epilepsy (PSE) are clinically important complications after acute ischemic stroke. DVT commonly occurs in patients with greater neurological impairment, immobility, inflammation, and hypercoagulability, factors that may also be associated with PSE. Whether DVT is independently associated with PSE remains uncertain.

**Methods:**

We conducted a secondary analysis of a multicenter cohort of 21,459 patients with acute ischemic stroke followed for 1 year. The association between recorded DVT and PSE was examined using sequential logistic regression models adjusting for demographic characteristics, vascular comorbidities, stroke severity, and lesion phenotype. Multicollinearity, sensitivity analyses, subgroup analyses, and joint stratification by DVT and National Institutes of Health Stroke Scale (NIHSS) score were performed. The incremental predictive value of DVT was evaluated using the area under the receiver operating characteristic curve (AUC), Brier score, calibration, and decision curve analysis.

**Results:**

Among 21,459 patients, 1,324 (6.2%) had DVT and 936 (4.4%) developed PSE. DVT was associated with higher odds of PSE in the unadjusted analysis (OR, 1.93; 95% CI, 1.56–2.39), and the association remained similar after adjustment for demographic and vascular factors. After further adjustment for NIHSS score, cortical lobe involvement, and anterior and posterior circulation involvement, the association was substantially attenuated and no longer statistically significant (OR, 1.10; 95% CI, 0.86–1.39; *P* = 0.45). Results were consistent across sensitivity analyses. Adding DVT to the clinical model did not improve discrimination (AUC, 0.879 vs. 0.879; DeLong *P* = 0.255), Brier score, calibration, or decision-curve net benefit.

**Conclusions:**

The association between DVT and 1-year PSE was largely explained by stroke severity and lesion phenotype. DVT provided little incremental predictive information beyond established clinical characteristics, supporting its interpretation as a marker of a more severe and clinically complex poststroke phenotype rather than an independent PSE risk factor.

## 1 Introduction

Poststroke epilepsy (PSE) is an important long-term neurological complication of acute ischemic stroke and can adversely affect functional recovery, quality of life, and long-term clinical management (1–3). Identifying patients at increased risk of PSE is therefore clinically relevant for individualized follow-up and early risk stratification (4–6). Previous studies have consistently identified greater stroke severity and cortical involvement as important clinical determinants of PSE, while accumulating evidence suggests that inflammatory responses, blood-brain barrier disruption, and coagulation-related pathways may also contribute to poststroke epileptogenesis (7–10).

Deep vein thrombosis (DVT) is a common complication of acute stroke and develops in the setting of venous stasis, endothelial dysfunction, hypercoagulability, and inflammation (11–14). Inflammation and coagulation are closely interconnected, with inflammatory activation promoting thrombosis and thrombotic processes in turn capable of amplifying inflammatory signaling (15, 16). Among patients with ischemic stroke, DVT and venous thromboembolism occur more frequently in those with greater neurological impairment, leg weakness, reduced mobility, larger infarct burden, prolonged hospitalization, and elevated inflammatory or coagulation markers such as C-reactive protein and D-dimer (17–20). These characteristics overlap substantially with the clinical profile of patients at increased risk of PSE.

This overlap raises an important clinical question: whether DVT is associated with subsequent PSE independently of the underlying severity and phenotype of ischemic stroke, or whether it primarily reflects a more severe and thromboinflammatory poststroke state. However, the relationship between DVT and PSE has not been well characterized. We therefore examined the association between recorded DVT and 1-year PSE in a large multicenter cohort of patients with acute ischemic stroke. We further evaluated whether the association persisted after accounting for demographic characteristics, vascular comorbidities, stroke severity, and lesion phenotype, and whether DVT provided incremental predictive information beyond these established clinical factors.

## 2 Methods

### 2.1 Study design and population

We conducted a secondary analysis of a publicly available, deidentified multicenter cohort of adults hospitalized with acute ischemic stroke. The source dataset included patients treated at 4 hospitals in Chongqing, China, between 2017 and 2023, with follow-up for 1 year. Patients aged 18 to 90 years with acute ischemic stroke, including lacunar infarction, were eligible (21). Patients were excluded if they had a history of stroke or transient ischemic attack, preexisting epilepsy or antiseizure medication use, neurologic disorders that could independently cause seizures, in-hospital or early poststroke death, loss to follow-up, or missing essential data. The final analytic cohort comprised 21,459 patients.

The source study received ethics approval from the participating institutions. Because the present study used deidentified, publicly available data, additional institutional review board approval and informed consent were not required.

### 2.2 Exposure and outcome

The exposure of interest was DVT, recorded as a binary comorbidity or complication in the source dataset. The publicly available data did not provide detailed information regarding the diagnostic modality, systematic vs. symptom-triggered screening, anatomical location, or timing of DVT relative to stroke onset. DVT was therefore analyzed according to the classification provided in the source dataset.

The primary outcome was PSE within 1 year, corresponding to the secondary epilepsy outcome recorded in the source dataset. Follow-up in the source study was based on outpatient records and telephone contact. Detailed information regarding seizure timing, electroencephalographic confirmation, physician adjudication, and the distinction between acute symptomatic seizures and late unprovoked seizures was unavailable. The outcome was therefore analyzed according to the original classification as a binary 1-year end point. Time-to-event analysis was not possible because the timing of PSE onset was unavailable.

### 2.3 Covariates and directed acyclic graph

Covariates were selected according to their clinical and biological relationships with DVT and PSE rather than according to univariable *P* values. Demographic and vascular variables included age, sex, hypertension, diabetes mellitus, hyperlipidemia, atrial fibrillation, coronary artery disease, and large-artery atherosclerosis. Stroke-related variables included admission National Institutes of Health Stroke Scale (NIHSS) score, cortical lobe involvement score, anterior circulation involvement, and posterior circulation involvement. The cortical lobe involvement score ranged from 0 to 5 according to involvement of the frontal, parietal, temporal, occipital, and insular lobes.

A conceptual directed acyclic graph was constructed to guide covariate selection and interpretation. Stroke severity and lesion phenotype were considered upstream factors potentially associated with both DVT and PSE. Immobility was considered an upstream determinant of DVT rather than a mediator of a DVT-PSE association. Acute symptomatic seizures and hemorrhagic transformation were represented as established but unavailable PSE-related factors, whereas systemic inflammation was considered only a hypothesized pathway potentially linking DVT with PSE.

### 2.4 Statistical analysis

Baseline characteristics were summarized according to DVT status. Continuous variables are presented as median (IQR) and were compared using the Wilcoxon rank-sum test. Categorical variables are presented as numbers (percentages) and were compared using the χ^2^ test or Fisher exact test, as appropriate (22, 23). Absolute standardized mean differences (SMDs) were additionally calculated to quantify between-group imbalance, with an SMD of 0.10 or greater considered potentially meaningful.

The association between DVT and 1-year PSE was evaluated using logistic regression, with results reported as odds ratios (ORs) and 95% CIs. Four sequential models were prespecified. Model 1 was unadjusted. Model 2 was adjusted for age and sex. Model 3 additionally included hypertension, diabetes mellitus, hyperlipidemia, atrial fibrillation, coronary artery disease, and large-artery atherosclerosis. Model 4 further included admission NIHSS score, cortical lobe involvement score, anterior circulation involvement, and posterior circulation involvement. NIHSS score and cortical lobe involvement score were modeled continuously. Additional laboratory and imaging variables were included in Table 1 for descriptive characterization but were not included in the adjustment models unless they were prespecified on clinical and biological grounds as potential confounders. Multicollinearity in Model 4 was assessed using variance inflation factors, with values greater than 5 considered indicative of potentially important multicollinearity.

**Table 1:** Baseline characteristics of the study population.

Variable	No DVT	DVT	*P*	SMD
Age, years	66.00 (59.00, 75.00)	74.00 (65.00, 81.00)	< 0.001	0.497
NIHSS score	7.00 (6.00, 9.00)	10.00 (8.00, 12.00)	< 0.001	0.783
Cortical lobe involvement score	0.00 (0.00, 0.00)	0.00 (0.00, 0.00)	< 0.001	0.248
Platelet count, 10^9^/L	187.80 (174.80, 203.60)	182.80 (170.10, 198.33)	< 0.001	0.180
White blood cell count, 10^9^/L	8.10 (7.50, 9.00)	8.70 (8.00, 9.70)	< 0.001	0.299
Red blood cell count, 10^12^/L	4.30 (4.10, 4.50)	4.20 (4.00, 4.30)	< 0.001	0.470
Hemoglobin A1c	6.40 (6.00, 7.20)	6.70 (6.30, 7.20)	< 0.001	0.178
C-reactive protein, mg/dL	8.90 (4.50, 17.80)	20.55 (10.78, 40.78)	< 0.001	0.520
Triglycerides, mmol/L	1.50 (1.30, 1.80)	1.40 (1.20, 1.60)	< 0.001	0.329
LDL cholesterol, mmol/L	2.70 (2.50, 2.90)	2.60 (2.40, 2.90)	< 0.001	0.105
HDL cholesterol, mmol/L	1.20 (1.20, 1.30)	1.30 (1.20, 1.30)	< 0.001	0.110
Serum albumin, g/L	41.30 (39.70, 42.50)	39.60 (37.58, 41.10)	< 0.001	0.736
Serum creatinine, μmol/L	75.00 (66.30, 86.50)	79.00 (72.20, 89.62)	< 0.001	0.106
D-dimer, mg/L	0.88 (0.64, 1.37)	1.99 (1.52, 2.82)	< 0.001	0.753
Fibrinogen, g/L	3.60 (3.30, 3.80)	3.80 (3.50, 4.20)	< 0.001	0.397
Sex	10,216 (50.74%)	627 (47.36%)	0.019	0.068
Hypertension	13,762 (68.35%)	989 (74.70%)	< 0.001	0.141
Diabetes mellitus	6,820 (33.87%)	502 (37.92%)	0.003	0.084
Hyperlipidemia	4,254 (21.13%)	215 (16.24%)	< 0.001	0.126
Atrial fibrillation	1,747 (8.68%)	296 (22.36%)	< 0.001	0.385
Coronary artery disease	8,970 (44.55%)	702 (53.02%)	< 0.001	0.170
Large-artery atherosclerosis	5,143 (25.54%)	362 (27.34%)	0.156	0.041
Anterior circulation involvement	1,265 (6.28%)	148 (11.18%)	< 0.001	0.174
Posterior circulation involvement	4,312 (21.42%)	189 (14.27%)	< 0.001	0.187
Any cortical lobe involvement	1,159 (5.76%)	170 (12.84%)	< 0.001	0.246
Frontal lobe involvement	793 (3.94%)	126 (9.52%)	< 0.001	0.224
Parietal lobe involvement	571 (2.84%)	92 (6.95%)	< 0.001	0.192
Temporal lobe involvement	545 (2.71%)	91 (6.87%)	< 0.001	0.196
Occipital lobe involvement	343 (1.70%)	52 (3.93%)	< 0.001	0.135
Insular lobe involvement	226 (1.12%)	53 (4.00%)	< 0.001	0.183

Continuous variables are presented as median (IQR) and categorical variables as No. (%). SMD indicates standardized mean difference.

DVT, deep vein thrombosis; HDL, high-density lipoprotein; LDL, low-density lipoprotein; NIHSS, national institutes of health stroke scale; SMD, standardized mean difference.

Three sensitivity analyses were performed by repeating the fully adjusted analysis after separately excluding patients with any cortical lobe involvement, patients with NIHSS scores greater than 15, and patients with large-artery atherosclerosis. Variables rendered invariant by the corresponding restriction were omitted from that sensitivity model.

Exploratory subgroup analyses were conducted according to age (< 65 vs. ≥65 years), sex, diabetes mellitus, atrial fibrillation, and NIHSS score (0–15 vs. 16–42). The NIHSS cutoff of 15 was selected based on commonly used severity categories, in which scores of 5 to 15 represent moderate stroke and scores above 15 indicate moderate-to-severe or severe neurological deficits (24, 25). For each subgroup, both unadjusted and Model 4–adjusted associations were estimated. Multiplicative interaction terms were tested using likelihood ratio tests. Because multiple subgroup comparisons were performed, interaction findings were considered exploratory.

For descriptive joint stratification, patients were categorized into 4 groups according to DVT status and NIHSS score (0–15 vs. 16–42), and the number and proportion developing PSE were reported for each group. NIHSS remained continuous in the primary regression models.

### 2.5 Incremental predictive value of DVT

An exploratory incremental prediction analysis was performed to determine whether DVT provided additional information beyond established clinical and stroke-related factors. A clinical model containing all Model 4 covariates except DVT was compared with the same model after addition of DVT.

Discrimination was evaluated using the area under the receiver operating characteristic curve (AUC) with 95% CIs, and AUCs were compared using the paired DeLong test. Overall prediction error was assessed using the Brier score. Calibration was evaluated by comparing predicted and observed event probabilities across deciles of predicted risk. Nested models were also compared using a likelihood ratio test, and decision curve analysis was used to evaluate net benefit across threshold probabilities from 1% to 20%.

Because predictive performance was evaluated in the same cohort used for model estimation, these analyses represent apparent performance and were intended to assess the incremental information provided by DVT rather than to develop or validate a new prediction model.

All tests were 2-sided, and *P* < 0.05 was considered statistically significant. No adjustment for multiple comparisons was applied to exploratory subgroup analyses. Statistical analyses were performed using R 4.4.1 (R Foundation for Statistical Computing).

## 3 Results

### 3.1 Baseline characteristics of the study population

A total of 21,459 patients with acute ischemic stroke were included, of whom 1,324 (6.2%) had recorded DVT and 936 (4.4%) developed PSE within 1 year. Compared with patients without DVT, those with DVT were older (median age, 74 vs. 66 years), had greater neurological severity (median NIHSS score, 10 vs. 7), and more frequently had cortical involvement, atrial fibrillation, coronary artery disease, and anterior circulation involvement. Patients with DVT also had higher C-reactive protein and D-dimer levels and lower serum albumin levels. The largest standardized differences were observed for NIHSS score (SMD, 0.783), D-dimer (SMD, 0.753), serum albumin (SMD, 0.736), C-reactive protein (SMD, 0.520), age (SMD, 0.497), and red blood cell count (SMD, 0.470). In contrast, sex, diabetes mellitus, and large-artery atherosclerosis showed relatively small between-group differences (Table 1).

### 3.2 Association between DVT and PSE

The conceptual relationships among DVT, stroke severity, lesion phenotype, and PSE are shown in Figure 1A. In the unadjusted analysis, DVT was associated with higher odds of 1-year PSE (OR, 1.93; 95% CI, 1.56–2.39). The association remained essentially unchanged after adjustment for age and sex (OR, 2.10; 95% CI, 1.69–2.60) and after additional adjustment for vascular comorbidities and large-artery atherosclerosis (OR, 1.98; 95% CI, 1.59–2.47). However, after further adjustment for admission NIHSS score, cortical lobe involvement, and anterior and posterior circulation involvement, the association was markedly attenuated and was no longer statistically significant (OR, 1.10; 95% CI, 0.86–1.39; *P* = 0.45; Figure 1B). There was no evidence of meaningful multicollinearity in the fully adjusted model, with VIF values ranging from 1.048 to 1.479 (Supplementary Table S1). The findings were consistent across the prespecified sensitivity analyses. DVT was not independently associated with PSE after excluding patients with any cortical lobe involvement (OR, 1.04; 95% CI, 0.80–1.35; *P* = 0.77), patients with NIHSS scores greater than 15 (OR, 0.80; 95% CI, 0.63–1.03; *P* = 0.09), or patients with large-artery atherosclerosis (OR, 1.19; 95% CI, 0.91–1.56; *P* = 0.20; Figure 1B).

**Figure 1:**
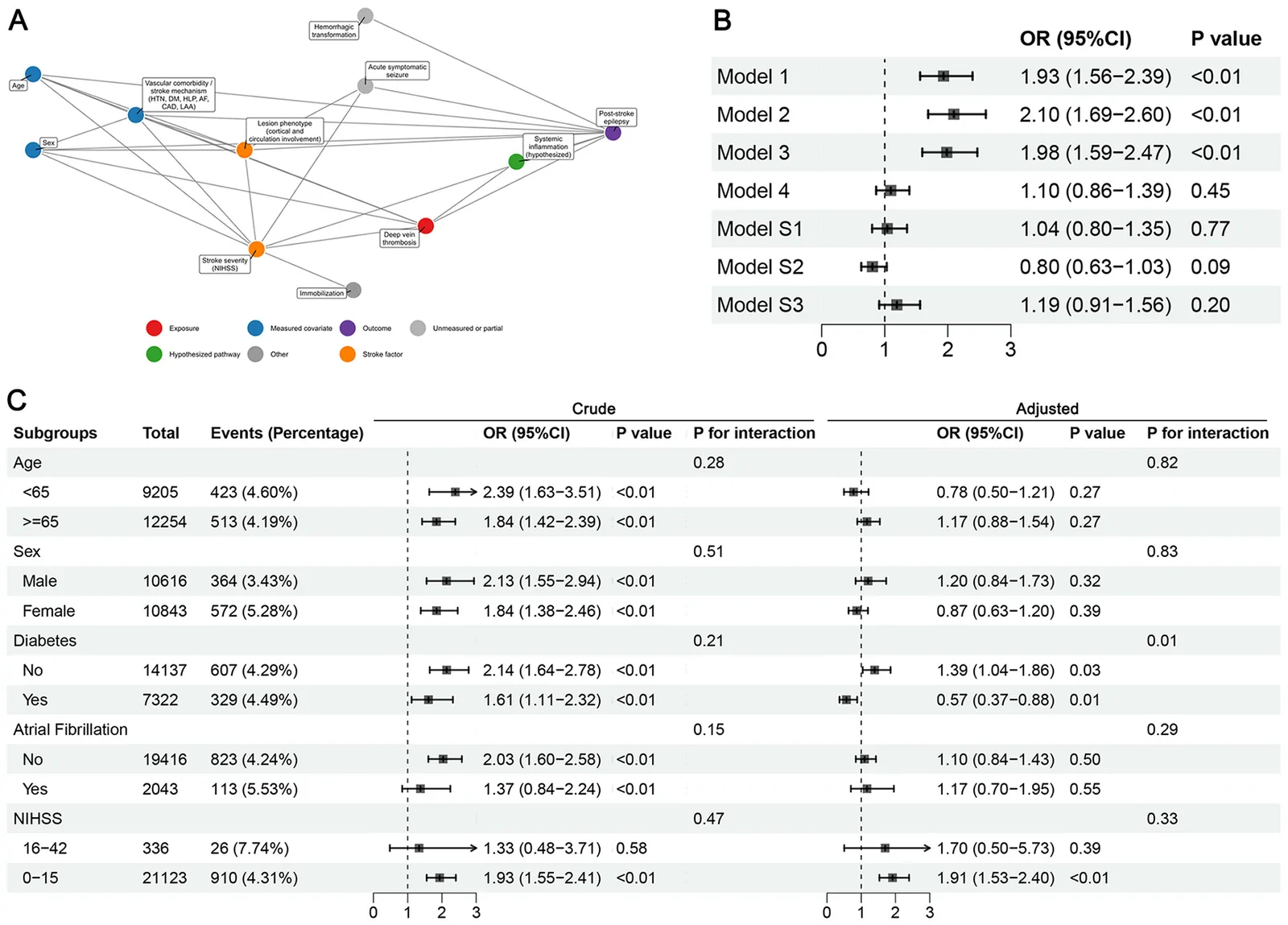
Association between deep vein thrombosis and poststroke epilepsy. **(A)** Conceptual directed acyclic graph of the hypothesized relationships between DVT and PSE. **(B)** Associations between DVT and PSE in sequentially adjusted models and sensitivity analyses. Model S1 excluded patients with cortical involvement; Model S2 excluded those with NIHSS scores greater than 15; and Model S3 excluded those with large-artery atherosclerosis. **(C)** Crude and fully adjusted subgroup analyses. Error bars indicate 95% CIs. DVT, deep vein thrombosis; NIHSS, national institutes of health stroke scale; OR, odds ratio; PSE, poststroke epilepsy.

### 3.3 Subgroup and joint stratified analyses

In crude subgroup analyses, DVT was generally associated with higher odds of PSE across most strata (Figure 1C). After Model 4 adjustment, most subgroup-specific associations were attenuated, with no evidence of interaction according to age, sex, atrial fibrillation, or NIHSS category (all *P* for interaction >0.05). A nominal interaction was observed according to diabetes status (*P* for interaction = 0.01): DVT was associated with higher odds of PSE among patients without diabetes (OR, 1.39; 95% CI, 1.04–1.86) but with lower odds among those with diabetes (OR, 0.57; 95% CI, 0.37–0.88). Given the exploratory nature of the subgroup analyses and multiple comparisons, this finding should be interpreted cautiously.

In the joint descriptive analysis using an NIHSS cutoff of 15, PSE occurred in 813 of 19,851 patients (4.10%) with NIHSS scores of 15 or lower and no DVT and in 97 of 1,272 (7.63%) with NIHSS scores of 15 or lower and DVT. Among patients with NIHSS scores greater than 15, PSE occurred in 21 of 284 patients (7.39%) without DVT and 5 of 52 (9.62%) with DVT (Table 2).

**Table 2:** One-year PSE across joint NIHSS and DVT groups.

NIHSS > 15	DVT	*N*	Epilepsy
No	No	19,851	813 (4.10%)
No	Yes	1,272	97 (7.63%)
Yes	No	284	21 (7.39%)
Yes	Yes	52	5 (9.62%)

Data are presented as No. (%) of patients with PSE. NIHSS was categorized as 0–15 and 16–42.

DVT, deep vein thrombosis; NIHSS, national institutes of health stroke scale; PSE, poststroke epilepsy.

### 3.4 Incremental predictive value of DVT

The clinical model incorporating demographic, vascular, stroke severity, and lesion-related characteristics showed good discrimination for 1-year PSE (AUC, 0.879; 95% CI, 0.868–0.890). Addition of DVT did not improve discrimination, with an identical AUC of 0.879 (95% CI, 0.868–0.890; DeLong *P* = 0.255; Figure 2A). Overall prediction error was also essentially unchanged, with a Brier score of 0.038 for both models. Calibration patterns were nearly overlapping between the clinical model and the model additionally including DVT (Figure 2B).

**Figure 2:**
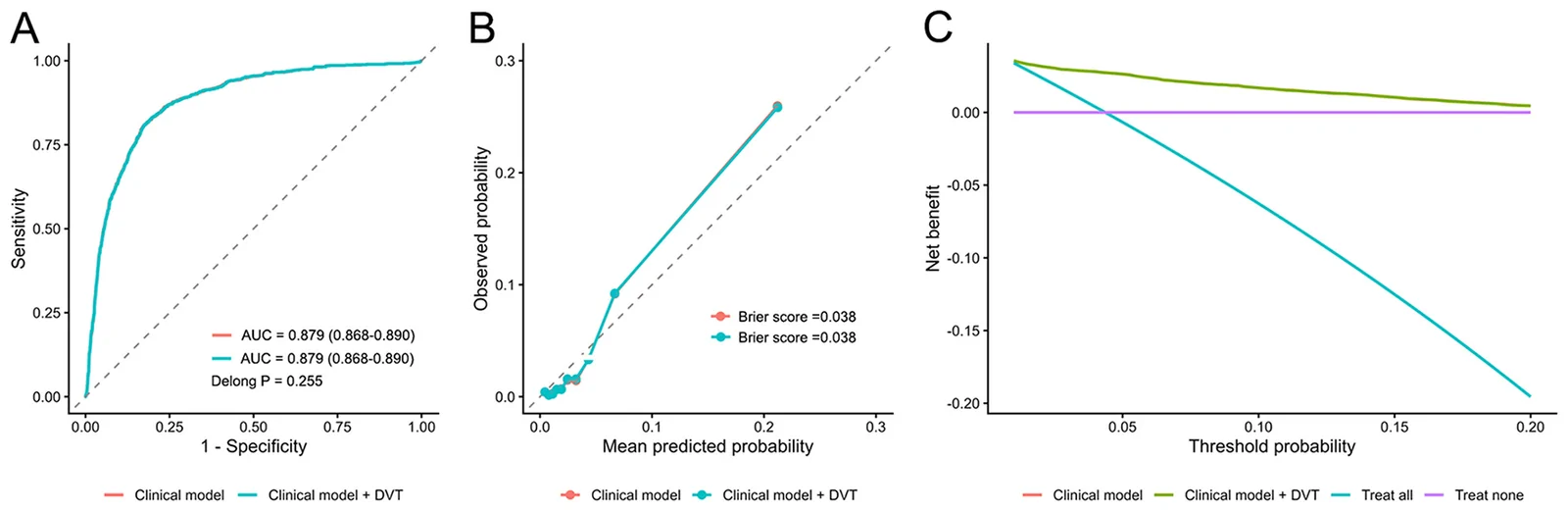
Incremental predictive value of deep vein thrombosis for poststroke epilepsy. **(A)** Receiver operating characteristic curves for the clinical model with and without DVT. **(B)** Calibration plots with corresponding Brier scores. **(C)** Decision curve analysis across threshold probabilities of 1% to 20%. AUC, area under the receiver operating characteristic curve; DVT, deep vein thrombosis; PSE, poststroke epilepsy.

Decision curve analysis similarly showed no appreciable incremental net benefit from adding DVT across the evaluated threshold probabilities (Figure 2C). Collectively, these findings suggest that DVT provided little additional predictive information for PSE beyond established clinical, stroke severity, and lesion-related characteristics.

## 4 Discussion

In this large multicenter cohort of patients with acute ischemic stroke, recorded DVT was associated with approximately 2-fold higher odds of PSE within 1 year in the unadjusted model, and this association remained largely unchanged after adjustment for demographic characteristics and vascular comorbidities. However, the association was markedly attenuated after further accounting for admission NIHSS score, cortical involvement, and anterior and posterior circulation involvement and was no longer statistically significant. The findings were consistent in sensitivity analyses excluding patients with cortical involvement, NIHSS scores greater than 15, or large-artery atherosclerosis. Moreover, adding DVT to a clinical model containing demographic, vascular, stroke severity, and lesion-related characteristics did not improve discrimination, overall prediction error, calibration, or clinical net benefit. Taken together, these findings suggest that the observed association between DVT and PSE is largely attributable to the underlying severity and phenotype of ischemic stroke rather than to an independent association of DVT with subsequent epilepsy.

The substantial attenuation after inclusion of stroke severity and lesion characteristics is clinically plausible. Stroke severity and cortical involvement are among the most consistently reported determinants of PSE. A recent systematic review and meta-analysis found substantially increased PSE risk among patients with severe stroke and cortical involvement, while anterior circulation infarction, hemorrhagic transformation, and acute symptomatic seizures were also associated with subsequent epilepsy (26). Reviews of PSE have similarly identified initial stroke severity and cortical injury as major clinical predictors (2). These same features may also increase the likelihood of DVT. Patients with more severe neurological deficits are more likely to have limb weakness, reduced mobility, prolonged hospitalization, infection, and greater systemic physiological disturbance. A systematic review of venous thromboembolism after ischemic stroke identified prestroke disability, leg weakness, increasing infarct volume, infection, longer hospitalization, elevated D-dimer, and elevated C-reactive protein among factors associated with VTE risk (18), consistent with earlier prospective observations linking neurological impairment and immobility with poststroke DVT (17). Thus, DVT and PSE may share important upstream determinants, particularly the extent and clinical consequences of the initial cerebral injury.

A biological relationship between thrombosis and epileptogenesis nevertheless remains plausible and warrants further investigation. Venous thrombosis develops through interactions among venous stasis, endothelial activation, coagulation, and inflammation. Increasing evidence supports a bidirectional relationship between inflammation and venous thrombosis, involving endothelial cells, platelets, leukocytes, cytokines, and coagulation pathways (19, 27, 28). In parallel, neuroinflammation, blood-brain barrier dysfunction, and coagulation-related signaling have been implicated in poststroke epileptogenesis. Blood-brain barrier disruption can promote neuroinflammation, edema, and exposure of brain tissue to circulating factors that may enhance neuronal hyperexcitability, while thrombin and protease-activated receptor signaling have also been proposed as contributors to maladaptive plasticity after ischemic injury (10, 29). These overlapping mechanisms provide biological grounds for investigating DVT in relation to PSE. However, the present results do not support a strong independent association once stroke severity and lesion phenotype are considered. Rather than demonstrating that DVT itself promotes epileptogenesis, our findings are more consistent with DVT identifying patients in whom severe neurological injury, immobility, inflammation, and coagulation activation coexist.

The incremental prediction analyses further clarify the potential clinical role of DVT. The clinical model showed good discrimination for 1-year PSE, with an AUC of 0.879, but addition of DVT left the AUC essentially unchanged and did not significantly improve discrimination. Brier scores were also nearly identical, and the calibration and decision curves showed no meaningful improvement after DVT was added. These findings are important because an observed unadjusted association does not necessarily imply that a variable contributes useful prognostic information beyond established clinical predictors. In the present study, DVT was readily identifiable and was associated with PSE before comprehensive adjustment, but it added little information once NIHSS and lesion characteristics were known. Accordingly, DVT should not be regarded as a stand-alone PSE risk-stratification variable on the basis of these data, nor do our findings support antiseizure prophylaxis or intensified seizure surveillance solely because DVT is present. Its clinical relevance may instead lie in signaling a more complicated poststroke course in which established PSE risk factors should be assessed carefully.

The subgroup analyses should be interpreted in this context. Most subgroup-specific associations were substantially attenuated after full adjustment, with no evidence of interaction according to age, sex, atrial fibrillation, or NIHSS category. An interaction according to diabetes status was observed in the fully adjusted analysis; however, the direction of association differed between strata, and multiple subgroup comparisons were performed without multiplicity adjustment. We therefore consider this finding exploratory and potentially attributable to chance rather than evidence of a reproducible biological interaction. Similarly, the joint analysis using an NIHSS cutoff of 15 was intended to provide a descriptive view of absolute PSE frequency across combinations of neurological severity and DVT status rather than to establish a threshold-dependent effect of DVT.

This study has several strengths. It included a large multicenter cohort, incorporated both neurological severity and lesion-related characteristics into the fully adjusted analysis, explicitly evaluated multicollinearity, and examined robustness across several clinically relevant sensitivity analyses. In addition, we directly assessed whether DVT provided incremental predictive information rather than inferring prognostic usefulness from statistical association alone. Several limitations should also be acknowledged. First, DVT was available only as a binary variable, and information regarding diagnostic modality, screening strategy, anatomical location, treatment, and timing relative to stroke onset was unavailable. The temporal sequence between DVT and PSE therefore cannot be established with precision. Second, detailed criteria for PSE diagnosis, seizure timing, electroencephalographic confirmation, and physician adjudication were unavailable, limiting our ability to distinguish acute symptomatic seizures from late unprovoked seizures and introducing the possibility of outcome misclassification. Third, several established PSE-related factors, including acute symptomatic seizures, hemorrhagic transformation, detailed lesion characteristics, and reperfusion treatment, were unavailable, and residual confounding cannot be excluded. Fourth, the timing of PSE onset was unavailable, precluding time-to-event analyses. Finally, the incremental prediction analyses were performed in the same cohort used for model estimation and therefore represent apparent performance rather than external validation. These predictive findings should consequently be interpreted as evidence regarding the incremental information provided by DVT, not as validation of a new PSE prediction model.

## 5 Conclusion

In this large multicenter cohort of patients with acute ischemic stroke, DVT was associated with higher odds of PSE in crude and partially adjusted analyses, but this association was substantially attenuated after accounting for stroke severity and lesion phenotype. The findings remained consistent across sensitivity analyses, and DVT provided little incremental predictive value beyond established clinical and stroke-related characteristics. These results suggest that DVT is better interpreted as a marker of a more severe and clinically complex poststroke phenotype than as an independent risk factor for PSE.

## Data Availability

Publicly available datasets were analyzed in this study. The data used in this secondary analysis are publicly available in the Dryad Digital Repository (doi: 10.5061/dryad.w0vt4b92c).
