# Supplementary material for: Association of deep vein thrombosis with poststroke epilepsy after acute ischemic stroke: a multicenter retrospective cohort study
Source: Front Neurol. 2026 Sep 25;17:1920544. doi: 10.3389/fneur.2026.1920544 (PMC13649490; doi:10.3389/fneur.2026.1920544)
Supplement: Supplementary file 1 [file Table_1.docx]

Table S1. Variance Inflation Factors for the Fully Adjusted Model

| Variable | VIF |
| --- | --- |
| Deep Vein Thrombosis | 1.048 |
| Age | 1.331 |
| Sex | 1.089 |
| Hypertension | 1.147 |
| Diabetes Mellitus | 1.130 |
| Hyperlipidemia | 1.070 |
| Atrial Fibrillation | 1.166 |
| Coronary Artery Disease | 1.273 |
| Large Artery Atherosclerosis | 1.114 |
| NIHSS Score | 1.479 |
| Cortical Lobe Involvement Score | 1.105 |
| Anterior Circulation Involvement | 1.133 |
| Posterior Circulation Involvement | 1.063 |

VIFs were calculated for variables included in Model 4 to assess multicollinearity.

Abbreviations: NIHSS, National Institutes of Health Stroke Scale; VIF, variance inflation factor.
